# The association between HIV infection, disability and lifestyle activity among middle-aged and older adults: an analytical cross-sectional study in Ivory Coast (the VIRAGE study)

**DOI:** 10.1186/s12889-024-19020-9

**Published:** 2024-06-08

**Authors:** Pierre Debeaudrap, Nadine Etoundi, Joseph Tegbe, Nelly Assoumou, Zelica Dialo, Aristophane Tanon, Charlotte Bernard, Fabrice Bonnet, Hortense Aka, Patrick Coffie

**Affiliations:** 1 Centre Population and Development (Ceped), French National Research Institute for Sustainable Development (IRD) and Paris University, Inserm ERL 1244, 45 Rue Des Saints Pères, 75006 Paris, France; 2 Infectious and Tropical Diseases Service (SMIT), Treichville Teaching Hospital, Abidjan, Côte d’Ivoire; 3 Programme PAC-CI, Treichville Teaching Hospital, Abidjan, Côte d’Ivoire; 4 Department of Dermatology and Infectiology, UFR Sciences Médicales, Université Félix Houphouët-Boigny, Abidjan, Côte d’Ivoire; 5 University of Bordeaux, National Institute for Health and Medical Research (INSERM) UMR 1219, Research Institute for Sustainable Development (IRD) EMR 271, Bordeaux Population Health Centre, Bordeaux, France; 6 Service de Médecine Interne Et Maladies Infectieuses, CHU de Bordeaux, Hôpital Saint-André, Bordeaux, France; 7 Department of Psychology, Felix Houphouet Boigny University, Abidjan, Côte d’Ivoire, Ivory Coast

**Keywords:** HIV, Aging, Disability, Physical activity

## Abstract

**Introduction:**

People living with HIV (PLWH) live longer and face new health challenges resulting from the confluence of chronic HIV infection and the natural effect of aging and comorbidities. However, there is a dearth of information on the long-term impact of HIV infection on the health and wellbeing of PLWH in sub-Saharan Africa. This research aimed to fill this gap by reporting on physical, functional and social outcomes among PLWH treated at a referral center in Abidjan, Ivory Coast, and comparing them with those of a control group.

**Methods:**

Body composition, functional capacity, sarcopenia, limitations in daily activities and social participation were assessed among 300 PLWH (aged ≥ 30 years) and 200 uninfected adults of similar age and sex. The associations between these outcomes and participants’ socioeconomic characteristics, HIV history and physical activity level were assessed using generalized additive models adjusted for age and sex.

**Results:**

The median age was 51 years, and the median antiretroviral therapy duration was 15 years. Compared to controls, PLWH reported higher levels of physical activity (*p* < 0.0001). They had a lower muscle index (adjusted *p* < 0.0001) and grip strength (adjusted *p* < 0.0001) but achieved similar performance on the 6-min walk test (6MWT, *p* = 0.2). Among PLWH, physical activity level was positively associated with better performance in the 6MWT (*p* = 0.006) and greater hand grip strength (*p* = 0.04). The difference in physical performance according to the level of physical activity appeared mainly after the age of 60. PLWH reported similar rates of activity limitations (*p* = 0.8), lower depression levels and greater scores for social functioning (*p* = 0.02).

**Conclusion:**

In this study, PLWH achieved high levels of physical activity, which may explain why they maintained good physical performance and social functioning despite having a higher risk of sarcopenia. These results have important implications for resource-limited health systems and show avenues for chronic care models.

**Trial registration:**

This study was registered on the ClinicalTrials.gov website (NCT05199831, first registration the 20/01/2022).

**Supplementary Information:**

The online version contains supplementary material available at 10.1186/s12889-024-19020-9.

## Introduction

With the scale-up of antiretroviral therapy (ART), the survival of people living with HIV (PLWH) has improved dramatically, and HIV infection has become a chronic disease [1]. However, the confluence of chronic infection with HIV over the long term combined with the natural effect of aging is associated with a number of health challenges [2]. Chronic inflammation and immune activation associated with HIV, ART toxicity and the persistent occurrence of opportunistic infections increase the risk of cardiovascular diseases and musculoskeletal and neurocognitive disorders, among others [3–8]. Sarcopenia, the loss of muscle mass and function, is an important risk factor for mobility limitations in older people. PLWH are at increased risk of earlier onset and more severe forms of sarcopenia [9, 10].

In resource-limited countries, the situation is compounded by factors such as malnutrition or opportunistic infections, which remain frequent and can increase the risk and severity of these chronic conditions [11, 12]. These disorders result in substantial functional limitations, which, in turn, can limit the social participation of PLWH and impair their quality of life [13–16].

According to available studies, between one- and three-quarters of PLWH receiving ART experience some form of activity limitation [17–23]. However, there is important heterogeneity across studies and a paucity of data from low-income countries where the majority of PLWH live. In a systematic review reporting on physical function (gait and walking tests) among PLWH in sub-Saharan Africa, similar performance between HIV-infected and uninfected participants was found in half (*n* = 3) of the 6 studies included, and worse performance was found in the remaining studies [24]. Different moderating factors can explain this heterogeneity, including lifestyle factors such as tobacco use, alcohol consumption or the practice of physical activity [25, 26]. The presence and severity of comorbidities, e.g., high blood pressure, obesity or diabetes, may also vary across settings and impact people’s physical function [27, 28]. Environmental factors also need to be considered to understand the observed variations in health outcomes. Of them, health care characteristics are key, as they are direct determinants of the medical management of chronic HIV infection [29, 30] but should be complemented with additional information on the socioeconomic, cultural and organizational characteristics of the environment.

Regarding abilities for independent living and/or social participation, it has been stressed in studies conducted in sub-Saharan Africa that a large proportion of PLWH report limitations [21, 31–37]. However, studies that include both HIV-infected and uninfected people are scarce, and it is therefore extremely difficult to determine to what extent chronic HIV infection contributes to disability independently of people’s age, sex and environment. In the SAGE project led by the WHO, which assessed the burden of chronic disease and disabilities among adults aged 50 or older from low-income countries, there was only weak evidence for a positive association between HIV status and disability measured with the WHO Disability Assessment Schedule (WHODAS) score after adjusting for age and sex [35, 36].

Therefore, it is important to collect more data on the impact of chronic HIV infection on people’s lives as they age, accounting for the context in which they live. This research aimed to fill this gap by reporting on physical, functional and social outcomes among PLWH receiving care in a reference HIV center in Abidjan, Ivory Coast, and comparing them with those of an uninfected control group. In addition, we examined how lifestyle and environmental factors as well as HIV and treatment factors influenced the study outcomes.

## Methods

### Study design and setting

This was a cross-sectional evaluation of PLWH followed up at the Department of Infectious Disease and Tropical Medicine (SMIT) of Treichville University Teaching Hospital, Abidjan, Ivory Coast and of uninfected adults (controls) of similar age and sex. The SMIT is a reference HIV center that provides care to 3 680 PLWH. It was one of the first HIV clinics in Ivory Coast to provide antiretroviral therapy along with comprehensive HIV care in the context of the “Initiative” [38]. Participants were enrolled from February to December 2021. Participants living with HIV were randomly selected from the ART dispensation list and contacted by a social worker who invited them to participate in this study if they were older than 30 years, had been receiving ART for at least one year and had no acute illness. HIV-uninfected controls were recruited from the population of blood donors visiting a center nearby and from their social network. They were included in this study if they had a recent (< 3 months) negative HIV test and no acute illness. Control selection was stratified by age and sex so that their age and sex distribution was similar to that of the PLWH included in this study.

### Assessment of disability

Participants’ disability was analyzed according to the International Classification of Functioning, Disability and Health (ICF) framework developed by the WHO, which distinguishes three dimensions of disability [39] (S1 Fig): body function and structure, activity limitations and social participation restriction. It also considers the health conditions as well as the environmental and contextual factors that influence the limitations in the different dimensions. In this study, the evaluation of the first dimension of disability (body function) was focused on sarcopenia. In addition, comorbidities (overweight, high blood pressure and hyperglycemia) and depressed feeling (as assessed through the PHQ-9 questionnaire) were also included in this dimension (see details below). The second dimension of disability (activity limitations) was explored through standardized functional tests measuring physical performance (e.g., gait speed, see below) and questionnaires assessing activity limitations as perceived by people in their actual environment. The third dimension (social participation) was assessed through questionnaires and qualitative interviews, but only quantitative results are reported in this article. The personal and contextual factors considered in the analysis included participants’ socioeconomic characteristics (education, economic resources), their reported use of alcohol, the presence of depression symptoms and their level of physical activity. In addition, the effect of HIV infection (duration and immunodepression extent) and its treatment (toxicities and failure) on the different outcomes was assessed. Treatment toxicity was defined as any switch in the ART combination due to adverse effect, while treatment failure was defined as switch in ART due to virologic failure, as reported in the participant’s medical file.

Mapping between the ICF conceptual framework and the different evaluations/instruments used and detailed below is provided in supplementary Fig. 1.

#### Assessment of sarcopenia

Three aspects of sarcopenia were distinguished: muscle mass, muscle strength and physical performance [40]. If isolated, low muscle mass corresponds to presarcopenia. Low muscle mass associated with low muscle strength corresponds to sarcopenia. Last, the combination of low muscle mass, low muscle strength and impaired physical performance corresponds to severe sarcopenia. The muscle index, computed as the skeletal muscle mass measured with bioimpedance analysis (BIA) divided by the square of height, was used to assess muscle mass. Muscle strength was assessed by the measurement of hand grip strength in kilograms using a digital handgrip dynamometer (JAMAR plus, Performance Health, France). Two measurements were taken from each hand, and the maximum of these was considered the outcome in the analysis.

Other clinical information was collected through a standardized physical evaluation. Height and weight were measured with the participants in light clothing and without shoes. Body mass index (BMI) was computed as the weight divided by the square of height, and participants with a BMI ≥ 30 kg/m^2^ were considered overweight. Blood pressure (BP) was measured using a digital monitor after resting for 10 min. Two measurements were taken, and the average was used in the analysis. High BP was defined as a systolic BP ≥ 140 or a diastolic BP ≥ 90 mm Hg. Glycated hemoglobin A1c (HbA1c) was measured using the HemoCue 501 assay and a cutoff of HbA1c ≥ 6.5% was used to identify participants with abnormal glucose metabolism. A composite indicator of comorbidities defined as the presence of at least one of the three comorbidities assessed (high blood pressure, overweight and abnormal glucose metabolism) was used in the analysis.

*Physical performance tests* included the 6-min walk test (6MWT), the 5 times sit-to-stand test (5STS), and the Short Physical Performance Battery (SPPB), which combines a balance test, a gait speed test and the 5STS. The 6MWT measures the distance covered during 6 min at the fastest comfortable pace. It assesses aerobic endurance and reflects global functional performance [41]. The 5STS measures the time required to complete five sit-to-stand-to-sit cycles at the fastest comfortable pace. It assesses lower limb power and speed and overall balance [42]. The SPPB assesses lower limb function and balance and results in a score ranging from 0 to 12 (12 indicating the highest degree of functioning) [43]. In this analysis, poor physical performance was defined as an SPPB score < 10. More details regarding these tests and their scoring are provided in the Supplementary Material.

#### Instrumental activities of daily living (IADLs) 

The Daily Functioning Interference scale (DFI) was used to measure IADLs. It includes 16 items that assess activities of daily living adapted to the African context [44] and allows direct mapping with the Lawton questionnaire [45], resulting in a score ranging from 0 to 8.

*The WHODAS-2* covers both limitations in daily activities and restrictions in social participation [46]. It also assesses the influence of the patient's environment on her functioning. This instrument has been used in different cultural contexts and has shown good validity and reliability [47]. In this study, the overall reliability coefficient was 0.75. The WHODAS-2 total score was computed using a weighted sum of the items and scaled to range from 0 (no disability) to 100 (extreme disability).

*Physical activity* was measured with the Global Physical Activity Questionnaire (GPAQ) [48, 49]. This instrument collects information on the practice of physical exercise in three situations (work, travel and leisure) and on sedentary behavior. The results are grouped into three categories: intense activity, moderate activity, and low activity. In addition, the perceptions of PLWH regarding having physical activity as part of their HIV care were assessed using Sidani et al.’s approach [50], which focuses on the following aspects of acceptability: relevance of the intervention to the person’s needs, adequacy with the person’s life, perceived efficacy, and ease of use.

*Depression* was assessed using the 9-item Patient Health Questionnaire (PHQ-9) depression scale [51]. The PHQ-9 is short and has very good sensitivity and specificity to detect severe depression and good validity and reliability. It is one of the most frequently used instruments in multicultural contexts. In this study, the reliability coefficient was 0.87. In addition, the short version of the Alcohol Use Disorders Identification Test [AUDIT] [52] was used to assess alcohol consumption. Social isolation related to HIV was assessed by rating participants living with HIV’s agreement with the following statement “I tend to isolate myself from others because I am HIV positive” on a 5-point Likert scale.

All questionnaires were pretested and adapted when needed. They were administered by an interviewer who was specifically trained to achieve better standardization and avoid literacy bias.

### Statistical methods

In the first step, descriptive analyses were conducted. Participants’ sociodemographic characteristics and outcomes were summarized using medians and interquartile ranges for continuous variables and frequencies and percentages for categorical variables. Summaries were computed overall and by subgroups defined by HIV status, sex and age (≤ 50 or > 50 years).

The distribution of the variables was examined using histograms and density plots. It was compared against a normal distribution using a quantile–quantile (QQ) plot. When needed, Box‒Cox transformation of the variables was considered to achieve a distribution closer to normality.

In a second step, differences in the outcomes (muscle index, strength, physical performance and IADLs) adjusted for age and sex between participants with and without HIV infection were assessed using generalized additive models (GAMs) to account for a nonlinear effect of age [53]. Such a nonlinear effect was tested by transforming age into a categorial variable with two groups (≤ 50 years and > 50 years). The choice of a cutoff at 50 years was based on the literature as well as on group sizes. GAMs adjusted for age and sex were also used to explore the association between these outcomes and (1) participants’ levels of physical activity separately in the HIV-infected and uninfected groups and (2) HIV infection and its treatment characteristics (duration, nadir CD4, virologic failure, and toxicity) in PLWH only. A similar analysis was performed for the WHODAS score using nonparametric regression models, as significant skewness and floor effects were observed in the total score distribution [54]. Moreover, the associations between IADLs and WHODAS scores and physical performance were assessed separately among HIV-infected and uninfected participants using regression analysis (parametric logistic and nonparametric models, respectively) adjusted for age, sex, education level and economic resources. To ease interpretation, the physical performance variables were transformed into categorical variables, and the lower quartile was compared with the other quartiles.

The sample size of this study was determined to estimate the prevalence of functional limitations and difficulties among PLWH with an accuracy ≥ 10% assuming an incomplete response rate up to 10% and to detect a prevalence ratio of functional limitations between the HIV-infected and uninfected groups ≥ 1.5. In this analysis, missing data were not imputed.

All data analyses were performed using R [55].

### Ethics

The VIRAGE study was approved by the “Comité National d’Ethique des Sciences de la Vie et de la Santé” in Ivory Coast (072–2/MSHP/CNESVS-kp). All participants gave written informed consent before inclusion. This study was registered on the ClinicalTrials.gov website (NCT05199831).

## Results

### Study population

A total of 300 PLWH (96 men and 204 women) and 200 adults without HIV infection (57 men and 143 women) were included in this study. Most participants living with HIV had received ART for more than 10 years (median: 15 years) and had CD4 cell counts at ART initiation below 200 cells/mm^3^ (Table 1). Participants with and without HIV infection had similar age and sex distributions (*p* = 0.8 and *p* = 0.5), and almost half of them were aged 50 or older (Table 1). There were some differences in the other sociodemographic characteristics between the two groups. Participants without HIV infection were more likely to have higher education levels (age- and sex-adjusted p value [p_as_]: 0.03) and a paid job (p_as_ < 0.0001), while those living with HIV were more likely to make their living through informal work (p_as_ < 0.0001). However, there was no significant difference in the reported economic resources between the two groups (p_as_ = 0.3).

**Table 1:** Characteristics of the study participants

	**Participants living with HIV**			**Participants without HIV infection**		
	**Men** (*n* = 96)	**Women** (*n* = 204)	**All** (*n* = 300)	**Men** (*n* = 57)	**Women** (*n* = 143)	**All** (*n* = 200)
Age, median (IQR)	54 (49–60)	49 (43–53)	50 (45–56)	52 (47–59)	52 (44–58)	52 (45–58)
Education level, n (%)						
None	12 (13)	43 (21.5)	55 (19)	2 (3.5)	27 (19)	29 (14.5)
Elementary	12 (13)	58 (29)	70 (24)	4 (7)	37 (25.9)	41 (20.5)
Secondary	38 (41)	73 (36.5)	111 (38)	35 (61)	57 (40)	92 (46)
Higher	30 (33)	26 (13)	56 (19)	16 (28)	22 (15)	38 (19)
Work status, n (%)						
Paid work or retired	74 (77)	80 (39)	154 (51)	48 (84)	95 (66)	143 (71.5)
Informal or home work	13 (13.5)	92 (45)	105 (35)	3 (5.3)	25 (17.5)	28 (14)
No work	9 (9)	32 (16)	41 (14)	6 (10.5)	23 (16)	29 (14.5)
Disposable income, n (%)						
Yes	44 (46)	104 (51)	148 (49)	32 (56)	74 (52)	106 (53)
Body mass index, n (%)						
≤ 25	65 (69)	77 (39)	142 (49)	29 (51)	33 (23)	62 (31)
25–30	20 (21)	69 (35)	89 (30)	21 (37)	48 (34)	69 (35)
> 30	9 (10)	52 (26)	61 (21)	7 (12)	62 (43)	69 (35)
High blood pressure, n (%)	35 (40)	48 (25)	79 (30)	21 (37)	47 (33)	68 (34)
HbA1c ≥ 6.5%, n (%)	7 (9)	17 (10)	24 (10)	8 (14)	22 (15)	30 (15)
Skeletal muscle index, median (IQR)	18 (17–19)	16 (15–17)	17 (15–18)	19 (17–19)	17 (15–18)	17(16–19)
Fat mass index, median (IQR)	18 (17–19)	16 (15–17)	17 (15–18)	19 (17–19)	17 (15–18)	17 (16–19)
Level of physical activity^a^						
High	33 (39)	52 (29)	85 (32)	11(19)	14 (10)	25 (12.5)
Moderate	31 (36)	63 (35.5)	94 (36)	28 (49)	54 (38)	82 (41)
Low	21 (25)	63 (35.5)	84 (32)	18 (32)	75 (52)	93 (46.5)
Audit score						
Low risk	78 (82)	169 (83)	247 (83)	29 (51)	125 (87)	154 (77)
Hazardous consumption^b^	4 (4)	18 (9)	22 (7)	10 (17)	4 (3)	14 (7)
Dependence^c^	13 (14)	16 (8)	29 (10)	18 (32)	14 (10)	32 (16)
PHQ-9 score						
< 10	81 (98)	167 (92)	248 (94)	52 (91)	120 (84)	172 (86)
10–19	1 (1.2)	13 (7.2)	14 (5.3)	5 (9)	21 (15)	26 (13)
> 19	1 (1.2)	1 (0.6)	2 (0.8)	0	1 (1)	1 (0.5)
Duration of ART (IQR), years						
	14 (7–16)	13 (8–15)	13 (8–16)	-	-	-
CD4 cell count at ART initiation						
median (IQR)	148 (49–269)	181 (71–300)	177 (61–296)	-	-	-
missing	37	70	107			
Most recent CD4 cell count						
median (IQR)	462 (331–564)	569 (368–752)	520 (343–731)	-	-	-
missing	19	29	48			

*HbA1C* Glycated hemoglobin

^a^Reported through the GPAQ (see the Methods section for more details)

^b^Score ≥ 4 for men and ≥ 3 for women

^c^Score ≥ 5 for men and ≥ 4 for women

PLWH reported lower alcohol consumption and fewer depression symptoms than their counterparts without HIV infection (p_as_ = 0.06 and 0.007, respectively). In addition, they reported higher levels of physical activity (p_as_ < 0.0001). Walking accounted for approximately half of the reported energy expenditure (52%), work activities accounted for 42%, and leisure activities accounted for less than 10%. The perceptions of PLWH regarding including physical activity as part of their care were positive, as most of them found such an intervention to be relevant and efficient (*n* = 246, 93%), and most of them were ready to engage in this activity (*n* = 206, 78%).

Regarding the comorbidities assessed in this study (Table 1), the proportion of participants with high blood pressure was similar between those with and those without HIV infection (p_as_ = 0.6). There was a trend toward a higher prevalence of HbA1c above the cutoff of 6.5% in the control group (15% versus 10%, *p* = 0.1), and more participants without HIV infection were overweight (p_as_ = 0.001).

### Impairment: Muscle mass, muscle strength and physical performance

Compared to uninfected participants, those living with HIV had a lower muscle index (p_as_ < 0.0001, Table 1) and lower grip strength (age-, sex- and BMI-adjusted *p* value < 0.0001, Table 2). The results of the physical function tests are given in Table 2. Most participants had an SPPB score within the “normal” range (*n* = 414/460 [90%] had an SPPB score ≥ 10), and the proportions of participants with suboptimal SPPB scores were similar in the two groups (9.3% [25/268] versus 10.9% [21/192] among participants with and without HIV, respectively, p_as_ = 0.7). The 5STS was the subtest of the SPPB with the highest failure rate. While there was no significant difference in the 5STS scores between participants with and without HIV among those younger than 50 years (p_as_ = 0.5), better performances were observed among participants living with HIV older than 50 years compared to their counterparts without HIV infection older than 50 years (p_as_ = 0.05). Participants with and without HIV infection achieved similar distances in the 6MWT (p_as_ = 0.2).

**Table 2:** Outcomes of the physical tests by HIV status, sex and age: median (interquartile range)

	**Participants living with HIV**			**Participants without HIV infection**			
	**Men**	**Women**	**All**	**Men**	**Women**	**All**	*p*-value*
6-min walk test distance (m)							0.2
Age: 30 to 50 years	459 (411–495)	390 (356–422)	402 (358–445)	401 (377–495)	389 (347–415)	391 (347–421)	
Age: > 50 years	436 (390–468)	366 (330–408)	388 (341–442)	394 (371–441)	355 (306–388)	376 (333–400)	
All	442 (397–473)	380 (345–420)	398 (354–444)	397 (371–453)	379 (334–408)	388 (347–415)	
missing	9	17	26	2	5	7	
Five times sit-to-stand test (sec)							0.06
Age: 30–50 years	12 (10–13)	11.5 (10–13)	12 (10–13)	12 (10.8–13)	12 (10–13)	12 (10–13)	
Age: > 50 years	11 (10–13)	12 (10–14)	11.5 (10–14)	13 (10–14)	14 (12–15)	13 (11–15)	
All	11 (10–13)	12 (10–13)	12 (10–13)	12 (10–14)	13 (11–14)	13 (10.5–14)	
Missing	3	10	13	1	2	3	
Grip strength (kg)							< 0.0001
BMI < 19	32 (31–40)	23 (20–28)	31 (23–33)	36 (33–38)	36 (33–38)	36 (33–38)	
BMI: 19–25	42 (36–48)	28 (23–32)	32 (26–41)	48 (38–56)	32 (28–38)	37 (32–46)	
BMI > 25	45 (38–54)	28 (23–33)	30 (24–36)	47 (38–59)	34 (30–38)	35 (30–40)	
All	42 (35–49)	28 (23–32)	31 (24–38)	47 (38–59)	33 (30–38)	36 (31–41)	
Missing	2	1	3	0	0	0	
Muscle Index							< 0.0001
Age: 30–50 years	18 (17–19)	16 (15–17)	17 (15–18)	18 (17–19)	17 (16–18)	17 (16–19)	
Age: > 50 years	18 (16–19)	16 (15–17)	17 (16–18)	19 (18–20)	16 (15–18)	17 (16–19)	
All	18 (17–19)	16 (15–17)	17 (15–18)	19 (17–19)	17 (15–18)	17 (16–19)	
Missing	3	6	9	0	0	0	

*BMI* Body mass index, *IQR* Interquartile range

*; HIV-infected versus uninfected adjusted for age and sex

### Association between physical performance, sarcopenia and physical activity

**Fig. 1:**
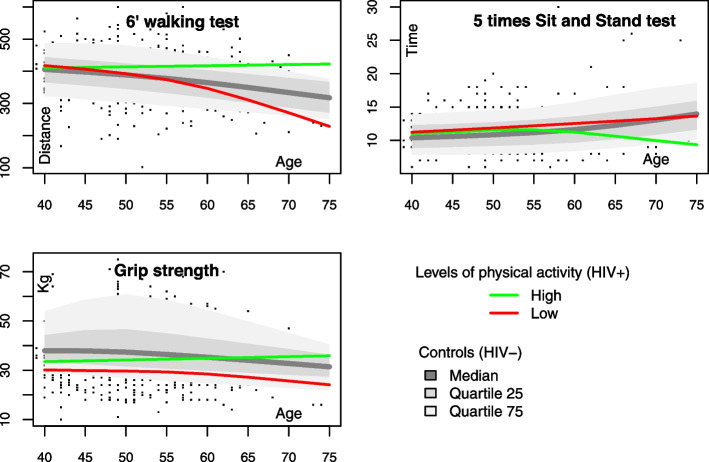
Shape of the association between physical capacities (6MWT, 5STS), grip strength and the skeletal muscle index and age and physical activity levels. Green and red lines represent the fitted outcomes using GAMs adjusted for age among PLWH who reported high physical activity (green) and low physical activity (red). The gray lines represent the 25%, 50% and 75% quantile estimates for HIV-uninfected participants overall. Note that lower 5STS time indicates greater physical capacity. kg: kilogram

A higher level of physical activity was associated with better performance in the 6MWT and greater hand grip strength among participants with HIV (p_as_ = 0.006 and 0.04, respectively) but not among those without HIV (all *p* values were > 0.3). As displayed in Fig. 1, the difference in physical performance between participants who reported a high level of physical activity and those who reported a low level appeared mainly after the age of 60 (*p* = 0.003 for the 6MWT and 0.07 for grip strength). Among participants living with HIV, there was a trend toward a positive association between performance on the 5STS and activity level (p_as_ = 0.1). In contrast, physical activity was not associated with the muscle index in either group (all *p* values were > 0.4).

### Association between physical performance and factors related to HIV infection characteristics

**Fig. 2:**
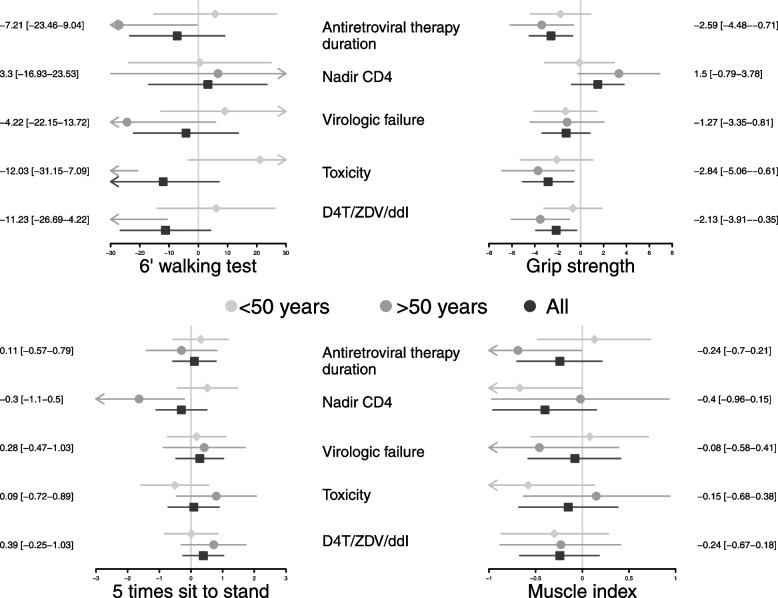
Association between physical performance outcomes (6MWT, 5STS, grip strength and muscle index) and factors related to HIV infection and treatment characteristics (ART duration, CD4 nadir, history of virologic failure, history of treatment with D4T, DDI or ZDV)

This analysis was restricted to participants living with HIV. As shown in Fig. 2, the variables related to HIV infection and its treatment were not associated with the 5STS or the muscle index. In contrast, ART duration, toxicity and history of treatment with didanosine (DDI), zidovudine (ZDV) and/or stavudine (D4T) were negatively associated with participants’ grip strength at all ages. Lower CD4 cell counts before ART and a history of virologic failure were also associated with decreased grip strength among those older than 50 years. Factors related to ART (i.e., duration, toxicity and a DDI/AZT/D4T-containing regimen) had a negative effect on 6MWT performance among older participants (> 50 years) but not among younger participants.

### Activity limitations

Compared to participants without HIV infection, a greater proportion of those living with HIV reported limitations in at least one daily activity (45% versus 33%, *p* = 0.04). However, it should be noted that the difference between the two groups was related exclusively to higher rates of difficulties reported by men living with HIV in meal preparation and household chores (Table 3). When these items were excluded for men as suggested by the authors of the IADL score [45], similar proportions of limitations were observed among PLWH compared to their uninfected peers (*p* = 0.8). Regarding participants’ social functioning assessed with the WHODAS-2 instrument, a higher level of difficulties was observed among uninfected participants compared to those living with HIV in all domains (all *p* < 0.05) but not the fourth (getting along with people, *p* = 0.8, Table 3).

**Table 3:** Activity limitation and disability (WHODAS-2) scores by sex and HIV status

	**Participants living with HIV**			**Participants without HIV infection**			
	**Men**	**Women**	**All**	**Men**	**Women**	**All**	*p*-value*
**Instrumental activities of daily living**							
Number of participants who could perform the activity independently^a^ (%)							
Ability to use telephone	84 (100)	181 (100)	265 (100)	55 (98)	141 (100)	196 (99.5)	0.4
Shopping	84 (100)	179 (99)	263 (99)	57 (100)	138 (97)	195 (98)	0.1
Food preparation	33 (39)	161 (89)	194 (73)	39 (75)	137 (96)	176 (90)	< 0.0001
Housekeeping	53 (63)	151 (83)	204 (77)	49 (88)	129 (90)	178 (89)	< 0.0001
Laundry	44 (52)	148 (82)	192 (73)	42 (74)	111 (78)	153 (77)	0.4
Mode of transportation	84 (100)	181 (100)	265 (100)	57 (100)	142 (99)	199 (99.5)	0.4
Responsibility for own medications	84 (100)	179 (99)	263 (99)	-	-	-	
Ability to handle finances	84 (100)	181 (100)	265 (100)	57 (100)	141 (99)	198 (99)	0.2
Median score (IQR)							
All activities	7 (6–7)	8 (7–8)	8 (7–8)	8 (7–8)	8 (7–8)	8 (7–8)	0.05
Restricted to 4 categories for men, 7 for women^b^	4 (4–4)	7 (6–7)		4 (4–4)	7 (6–7)		0.7
**WHODAS-2**							
Overall score, median (IQR)	0 (1.1–4.4)	1.1 (3.3–7.6)	0 (2.2–6.5)	2.2 (3.3–5.4)	3.3 (6.5–10.9)	2.7 (5.4–9.8)	< 0.0001
Missing	27	28	55	4	5	9	
Score by domain, median (IQR)							
Cognition	0 (0–10)	5 (0–10)	0 (0–10)	10 (0–15)	10 (5–15)	10 (0–15)	< 0.0001
Mobility	0 (0–6.2)	0 (0–12.5)	0 (0–6.2)	0 (0–6.2)	12.5(1.6–18.8)	6.2 (0–18.8)	< 0.0001
Self-care	0 (0–0)	0 (0–0)	0 (0–0)	0 (0–0)	0 (0–0)	0 (0–0)	< 0.0001
Getting along with people	0 (0–8.3)	0 (0–8.3)	0 (0–8.3)	0 (0–8.3)	8.3 (0–8.3)	0 (0–8.3)	0.8
Life activities	0 (0–0)	0 (0–10)	0 (0–0)	0 (0–0)	0 (0–17.5)	0 (0–10)	< 0.0001
Social participation	4.2 (0–8.3)	4.2 (0–13.5)	4.2 (0–12.5)	8.3 (4.2–12.5)	8.3 (4.2–19.8)	(4.2–16.7)	< 0.0001

^a^A score of 1 indicates independence, and a score of 0 (partial or complete) indicates dependence

^b^The category “Responsibility for medication” was excluded for men and women; the categories “food preparation”, “housekeeping” and “laundry” were excluded for men

*Participants with versus without HIV infection: exact test for categorical data and rank test for continuous data

In analyses adjusted for age, sex, education level and economic resources, the probability of reporting difficulties for at least one daily activity was associated with reduced 6MWT (impaired gait speed) among HIV-infected and uninfected participants (*p* = 0.04 and 0.03, respectively, Table 4). The WHODAS score (i.e., the level of difficulties) was higher among participants with impaired performance in the 6MWT in both HIV-infected and uninfected groups, although the magnitude of the associations was greater in the control group (Table 4). It was also associated with reduced grip strength and performance at the 5STS test among uninfected participants only. In the multivariate analysis adjusted for age, sex, education level, economic resources, grip strength and physical test performance, the mean WHODAS scores remained higher among uninfected participants than among those living with HIV (+ 2.43, 95% CI 1.60–3.26, *p* = 0.02). In the latter group, those who reported that they isolated themselves because of their HIV status had significantly higher (worse) WHODAS scores than other participants, whether they were uninfected (*p* < 0.0001) or infected (*p* = 0.0004).

**Table 4:** Association between physical performance outcomes and (1) the probability of reporting difficulties in at least one of the instrumental activities of daily living and (2) the WHODAS score. Physical performance outcomes were transformed into categorical variables using the lower quartile of the control group (or upper quartile for the 5STS test) as cutoff. All analyses were adjusted for age, sex and education level. The results for IADLs are odds ratios with 95% confidence intervals, while those for the WHODAS are the coefficients of linear regression with 95% confidence intervals

	**Univariate**		**Multivariate**	
	**HIV + **	**HIV-**	**HIV + **	**HIV-**
**Difficulties in Instrumental Activities of Daily Living**^**a**^				
Muscle index				
≤ 15.8 kg/m^2^ vs. > 15.8 kg/m^2^	1.17 (0.62 to 2.21)	1.16 (0.55 to 2.48)	0.65 (0.32 to 1.32)	0.84 (0.37 to 1.91)
6MWT				
≤ 347 m vs. > 347 m	2.55 (1.3 to 4.99)	4.26 (2.08 to 8.74)	**2.26 (1.03 to 4.96)**	**2.52 (1.10 to 5.77)**
5STS				
≤ 14 s vs. > 14 s	1.73 (0.77 to 3.86)	2.25 (1.03 to 4.92)	1.49 (0.59 to 3.78)	1.71 (0.69 to 4.26)
Grip strength				
≤ 31 kg vs. > 31 kg	2.35 (1.25 to 4.42)	2.64 (1.28 to 5.46)	0.95 (0.46 to 1.96)	1.72 (0.76 to 3.91)
Comorbidities				
High blood pressure and/or obesity and/or hyperglycemia	1 (0.52 to 1.93)	2.24 (1.03 to 4.87)	0.75 (0.35 to 1.64)	1.31 (0.55 to 3.13)
**WHODAS score** (higher score indicated increased level of difficulties)				
Muscle index				
≤ 15.8 kg/m^2^ vs. > 15.8 kg/m^2^	0 (-0.8 to 0.8)	0 (-1.93 to 1.93)	-0.22 (-1.26 to 0.83)	0 (-1.27 to 1.27)
6MWT				
≤ 347 m vs. > 347 m	**1.09 (0.05 to 2.12)**	**3.26 (1.31 to 5.21)**	**1.09 (0.32 to 1.86)**	**2.72 (1.48 to 3.96)**
5STS				
≤ 14 s vs. > 14 s	1.09 (-0.13 to 2.30)	2.17 (-0.03 to 4.37)	1.09 (-0.47 to 2.65)	**2.17 (0.1 to 4.25)**
Grip strength				
≤ 31 kg vs. > 31 kg	**1.09 (0.16 to 2.02)**	**4.35 (2.30 to 6.39)**	0.36 (-0.58 to 1.31)	**2.99 (1.26 to 4.72)**
Comorbidities				
High blood pressure and/or obesity and/or hyperglycemia	0 (-0.81 to 0.81)	**2.17 (0.40 to 3.95)**	0 (-1.07 to 1.07)	**1.36 (0.02 to 2.70)**

^a^Collected using the DFI questionnaire

## Discussion

In this study, we found that PLWH receiving ART and with good immunological status had overall good physical performance in reference to their peers without infection. These good outcomes could have resulted from the high level of physical activity observed in the population of this study. There is already evidence from clinical research showing the positive effect of physical activity on the health of PLWH and elderly individuals [25, 26]. However, empirical information from real life on the effect of physical activity remains scarce. Interestingly, although information on the uptake of physical activity in African contexts is limited, available studies indicate that most PLWH have low levels of physical activity, usually below what is recommended [56] or that of their peers without HIV infection [57]. Additional qualitative research is needed to identify the factors that have promoted this healthy behavior, which will help to design interventions for behavioral change and health promotion among PLWH.

The medical context is another aspect that could explain the good physical performance and health status of the participants living with HIV in this study. They were followed up at an HIV/AIDS reference center, and most of them initiated ART in the early 2000s, a period in which HIV care initiatives flourished. Participants in this study received emotional support in addition to the usual medical care and benefited from medical counseling and/or screening for other diseases, which is rarely possible for the rest of the population in such a context [58]. This has been noticed in the area of cancer, with greater coverage of cervical cancer screening found in some countries among women living with HIV compared to uninfected women [59]. This may explain why the participants living with HIV of this study reported limited consumption of alcohol and depressive symptoms, which contributes as well to the good outcomes observed in this population. Our results should therefore be interpreted taking into account the specific real-world context from which they come [60]. It is likely that this type of contextual factor explains a substantial part of the large heterogeneity observed between studies, and more effort should be made to report them in a systematic way.

Despite these encouraging results, we found that PLWH had impaired grip strength compared to their uninfected counterparts and that the difference appeared early. This is in line with available evidence showing that HIV infection accelerates the onset and enhances the progression of sarcopenia [10, 61, 62]. In addition, the negative association between grip strength and NRTI use found in this study is also consistent with a previous report indicating that NRTIs, especially zidovudine, have toxic effects on mitochondria [63]. It has also been shown that there is a poor correlation between grip strength and lower body strength as measured by the 5STS [64], which could explain the apparent discrepancy found between the lower grip strength observed in PLWH on the one hand and their good 5STS or 6MWT outcomes on the other. Our results suggest that physical activity helps to improve the strength of PLWH when they get older, which, in turn, may help to maintain good physical performance. As highlighted by Chetty and colleagues, more research is needed to determine the most effective training (type, frequency, duration and intensity) for older PLWH as well as how and when to implement physical activities in the management of HIV in a resource-limited context [65]. In this study population, we found that most of the physical activity was performed through walking and very little through training. However, participants reported that training with regular physical exercises could be an effective, feasible and acceptable intervention and ranked it high if it was conducted at their usual care center. It is now time to design participatory implementation and effectiveness studies to provide practical evidence to decision-makers.

Most published studies on disabilities among PLWH have focused on specific aspects of disabilities, either impairments, functional limitations or limitations in social functioning [66].

In this study, we attempted to provide a holistic perspective on disability by integrating physical and social functioning. We found similar levels of activity limitations yet better social functioning (as well as better mental health status) among participants living with HIV compared to their peers without infection. In addition, we found that the association between physical performance and WHODAS scores was twice as strong among uninfected participants than among those living with HIV, highlighting the multiple other factors that moderate the impact of impairment on day-to-day life and how it is perceived. People with chronic illness often adapt their expectations and references to cope with their new situation. As a consequence, a “response shift” in the evaluation of the daily difficulties can be observed with people having chronic condition reporting greater quality of life [67]. For participants living with HIV in this study, stigma appeared to be a more important determinant of social functioning than the physical limitations themselves. However, there may be a more complex relationship between stigma and disability, with each component enhancing the other. Moreover, a detailed analysis of stigma would be important in future research, distinguishing internalized and enacted stigma [68].

One of the strengths of this study lies in the inclusion of a control group of similar demographic characteristics allowing a better interpretation of the outcomes observed among the participants living with HIV. However, the control group was matched only on age and sex for practical reasons and the two groups differ with regard to other socio-economic characteristics. Although the analyses were adjusted on important factors such as age, sex, education level and financial resources, some unmeasured confounding may have remained. Other limitations of this study should be noted. First, the cross-sectional design of this study limited the analysis of the causal relation between HIV infection and physical activity on the one hand and the physical performance, disability and well-being of PLWH on the other hand. However, our results are consistent with those of other clinical studies [25, 26] and provide real-life information that will be useful for future research. Another limitation is the possibility of selection bias due to the non-inclusion of participants who did not come to take their drug because of health problems. It is also important to remember that participants in such surveys are patients who survived early mortality after HAART initiation [69]. There was more missing data for the disability questionnaire among participants living with HIV because the interviews had to be conducted during a second visit for practical reason. However, missingness was not associated with participants’ characteristics. This research was conducted after the first waves of the COVID epidemic. Various public health measures were introduced in Ivory Coast to limit the epidemic transmission, but it is unlikely that they had a major effect on the level of physical activity of the population as no home confinement was introduced.

In summary, this study shows that aging PLWH who have high levels of physical activity and receive adequate support can maintain good physical and social functioning. These results have important implications for resource-limited health systems and show avenues for chronic care models. Future research should assess the feasibility and effectiveness of implementing interventions in African contexts to promote and support engagement in physical activity among PLWH.

### Supplementary Information

Supplementary Material 1.

## Data Availability

The datasets analysed during the current study are available from the corresponding author on reasonable request.

## Abbreviations

PLWH: People living with HIV
6MWT: 6-Minute walk test
5STS: 5 Times sit-to-stand test
ddI: Didanosine
ZDV: Zidovuding
D4T: Stavudine

## References

[1] The Antiretroviral Therapy Cohort Collaboration (2008). Life expectancy of individuals on combination antiretroviral therapy in high-income countries: a collaborative analysis of 14 cohort studies. Lancet (London, England).

[2] Deeks SG, Lewin SR, Havlir DV (2013). The end of AIDS: HIV infection as a chronic disease. Lancet (London, England).

[3] Richert L, Brault M, Mercie P, Dauchy FA, Bruyand M, Greib C (2014). Decline in locomotor functions over time in HIV-infected patients. AIDS.

[4] Habib AG, Yakasai AM, Owolabi LF, Ibrahim A, Habib ZG, Gudaji M (2013). Neurocognitive impairment in HIV-1-infected adults in Sub-Saharan Africa: a systematic review and meta-analysis. International journal of infectious diseases : IJID : official publication of the International Society for Infectious Diseases.

[5] Robertson KR, Jiang H, Kumwenda J, Supparatpinyo K, Marra CM, Berzins B (2018). HIV associated neurocognitive impairment in diverse resource limited settings. Clin Infect Dis.

[6] Stringer WW (2000). Mechanisms of exercise limitation in HIV+ individuals. Med Sci Sports Exerc.

[7] Authier FJ, Chariot P, Gherardi RK (2005). Skeletal muscle involvement in human immunodeficiency virus (HIV)-infected patients in the era of highly active antiretroviral therapy (HAART). Muscle Nerve.

[8] Calligaro GL, Gray DM (2015). Lung function abnormalities in HIV-infected adults and children. Respirology.

[9] Natsag J, Erlandson KM, Sellmeyer DE, Haberlen SA, Margolick J, Jacobson LP (2017). HIV Infection Is Associated with Increased Fatty Infiltration of the Thigh Muscle with Aging Independent of Fat Distribution. PLoS ONE.

[10] Oliveira VHF, Borsari AL, Webel AR, Erlandson KM, Deminice R (2020). Sarcopenia in people living with the Human Immunodeficiency Virus: a systematic review and meta-analysis. Eur J Clin Nutr.

[11] Kerac M, Postels DG, Mallewa M, Alusine Jalloh A, Voskuijl WP, Groce N (2014). The interaction of malnutrition and neurologic disability in Africa. Semin Pediatr Neurol.

[12] Hestad KA, Chinyama J, Anitha MJ, Ngoma MS, McCutchan JA, Franklin DR (2019). Cognitive Impairment in Zambians With HIV Infection and Pulmonary Tuberculosis. J Acquir Immune Defic Syndr.

[13] Piggott DA, Erlandson KM, Yarasheski KE (2016). Frailty in HIV: Epidemiology, Biology, Measurement, Interventions, and Research Needs. Curr HIV/AIDS Rep.

[14] Rusch M, Nixon S, Schilder A, Braitstein P, Chan K, Hogg RS (2004). Prevalence of activity limitation among persons living with HIV/AIDS in British Columbia. Can J Public Health.

[15] Banks LM, Zuurmond M, Ferrand R, Kuper H (2015). The relationship between HIV and prevalence of disabilities in sub-Saharan Africa: systematic review (FA). Tropical medicine & international health : TM & IH.

[16] Rajasuriar R, Chong ML, Ahmad Bashah NS, Abdul Aziz SA, McStea M, Lee ECY (2017). Major health impact of accelerated aging in young HIV-infected individuals on antiretroviral therapy. AIDS.

[17] Myezwaa H, Hanass-Hancock J, Ajidahuna AT, Carpenter B (2018). Disability and health outcomes – from a cohort of people on long-term antiretroviral therapy. Journal of Social Aspects of HIV/AIDS.

[18] Greene M, Covinsky K, Astemborski J, Piggott DA, Brown T, Leng S (2014). The relationship of physical performance with HIV disease and mortality. AIDS.

[19] Laverick R, Haddow L, Daskalopoulou M, Lampe F, Gilson R, Speakman A (2017). Self-Reported Decline in Everyday Function, Cognitive Symptoms, and Cognitive Function in People With HIV. J Acquir Immune Defic Syndr.

[20] Van As M, Myezwa H, Stewart A, Maleka D, Musenge E (2009). The International Classification of Function Disability and Health (ICF) in adults visiting the HIV outpatient clinic at a regional hospital in Johannesburg. South Africa AIDS care.

[21] Hanass-Hancock J, Myezwa H, Carpenter B (2015). Disability and Living with HIV: Baseline from a Cohort of People on Long Term ART in South Africa. PLoS ONE.

[22] Hanass-Hancock J, Misselhorn A, Carpenter B, Myezwa H (2017). Determinants of livelihood in the era of widespread access to ART. AIDS Care.

[23] Keng LD, Winston A, Sabin CA (2023). The global burden of cognitive impairment in people with HIV. AIDS.

[24] Bernard C, Dabis F, de Rekeneire N (2017). Prevalence and factors associated with depression in people living with HIV in sub-Saharan Africa: A systematic review and meta-analysis. PLoS ONE.

[25] Robinson SM, Jameson KA, Syddall HE, Dennison EM, Cooper C, Aihie SA (2013). Clustering of lifestyle risk factors and poor physical function in older adults: the Hertfordshire cohort study. J Am Geriatr Soc.

[26] Zhou YF, Song XY, Pan XF, Feng L, Luo N, Yuan JM (2021). Association Between Combined Lifestyle Factors and Healthy Ageing in Chinese Adults: The Singapore Chinese Health Study. J Gerontol A Biol Sci Med Sci.

[27] Aubert CE, Kabeto M, Kumar N, Wei MY (2022). Multimorbidity and long-term disability and physical functioning decline in middle-aged and older Americans: an observational study. BMC Geriatr.

[28] Wei MY, Mukamal KJ (2018). Multimorbidity, Mortality, and Long-Term Physical Functioning in 3 Prospective Cohorts of Community-Dwelling Adults. Am J Epidemiol.

[29] Dawad S, Veenstra N (2007). Comparative health systems research in a context of HIV/AIDS: lessons from a multi-country study in South Africa, Tanzania and Zambia. Health Research Policy and Systems.

[30] Loveday M, Padayatchi N, Wallengren K, Roberts J, Brust JCM, Ngozo J (2014). Association between Health Systems Performance and Treatment Outcomes in Patients Co-Infected with MDR-TB and HIV in KwaZulu-Natal, South Africa: Implications for TB Programmes. PLoS ONE.

[31] Kietrys  D, Myezwa H, Galantino ML, Parrott JS, Davis T, Levin T (2019). Functional limitations and disability in persons living with HIV in South Africa and United States: similarities and differences. J Int Assoc Provid AIDS Care.

[32] Vancampfort D, Byansi PK, Namutebi H, Kinyanda E, Bbosa RS, Ward PB (2021). The efficacy of a lay health workers - led physical activity counselling program in patients with HIV and mental health problems: a real-world intervention from Uganda. AIDS Care.

[33] Cobbing S, Hanass-Hancock J, Myezwa H (2017). A Home-Based Rehabilitation Intervention for Adults Living With HIV: A Randomized Controlled Trial. J Assoc Nurses AIDS Care.

[34] Banda GT, Mwale G, Chimwala M, Malimusi L, Chisati E (2019). Common impairments and functional limitations of HIV sequelae that require physiotherapy rehabilitation in the medical wards at Queen Elizabeth Central Hospital, Malawi: A cross sectional study. Malawi Med J.

[35] Negin J, Martiniuk A, Cumming RG, Naidoo N, Phaswana-Mafuya  N, Madurai L (2012). Prevalence of HIV and chronic comorbidities among older adults. Aids.

[36] Mugisha JO, Schatz EJ, Randell M, Kuteesa M, Kowal P, Negin J (2016). Chronic disease, risk factors and disability in adults aged 50 and above living with and without HIV: findings from the Wellbeing of Older People Study in Uganda. Glob Health Action.

[37] Myezwa H, Hanass-Hancock J, Pautz N (2018). Investigating the interaction between human immunodeficiency virus, nutrition, and disability: A cross-sectional observational study. African journal of primary health care & family medicine.

[38] Msellati P, Vidal L, Moatti J (2001). L’accès aux traitementsdu VIH/sida en Côte d’Ivoire.

[39] World Health Organisation. International Classification of Functioning, Disability and Health (ICF). Geneva: World Health Organisation; 2011. Available from: http://www.who.int/icidh.

[40] Cruz-Jentoft AJ, Bahat G, Bauer J, Boirie Y, Bruyère O, Cederholm T (2019). Sarcopenia: revised European consensus on definition and diagnosis. Age Ageing.

[41] Steffen TM, Hacker TA, Mollinger L (2002). Age- and gender-related test performance in community-dwelling elderly people: Six-Minute Walk Test, Berg Balance Scale, Timed Up & Go Test, and gait speeds. Phys Ther.

[42] Lord SR, Murray SM, Chapman K, Munro B, Tiedemann A (2002). Sit-to-stand performance depends on sensation, speed, balance, and psychological status in addition to strength in older people. J Gerontol A Biol Sci Med Sci.

[43] Guralnik JM, Ferrucci L, Simonsick EM, Salive ME, Wallace RB (1995). Lower-extremity function in persons over the age of 70 years as a predictor of subsequent disability. N Engl J Med.

[44] Edjolo A, Peres K, Guerchet M, Pilleron S, Ndamba-Bandzouzi B, Mbelesso P (2019). Development of the Central Africa Daily Functioning Interference Scale for Dementia Diagnosis in Older Adults: The EPIDEMCA Study. Dement Geriatr Cogn Disord.

[45] Lawton MP, Brody EM (1969). Assessment of older people: self-maintaining and instrumental activities of daily living1. Gerontologist.

[46] Ustun TB, Kostanjsek N, Chatterjee S, Rehm J (2010). editors. Measuring health and disability: manual for WHO Disability Assessment Schedule (WHODAS 2.0).

[47] Federici S, Bracalenti M, Meloni F, Luciano  JV (2017). World Health Organization disability assessment schedule 2.0: An international systematic review. Disabil Rehabil.

[48] Bull FC, Maslin TS, Armstrong T (2009). Global physical activity questionnaire (GPAQ): nine country reliability and validity study. J Phys Act Health.

[49] Trinh OT, Nguyen ND, van der Ploeg HP, Dibley MJ, Bauman A (2009). Test-retest repeatability and relative validity of the Global Physical Activity Questionnaire in a developing country context. J Phys Act Health.

[50] Sidani S, Epstein DR, Bootzin RR, Moritz P, Miranda J (2009). Assessment of preferences for treatment: validation of a measure. Res Nurs Health.

[51] Kroenke K, Spitzer RL, Williams JB (2001). The PHQ-9: validity of a brief depression severity measure. J Gen Intern Med.

[52] Saunders JB, Aasland OG, Babor TF, de la Fuente JR, Grant M (1993). Development of the Alcohol Use Disorders Identification Test (AUDIT): WHO Collaborative Project on Early Detection of Persons with Harmful Alcohol Consumption–II. Addiction.

[53] Wood SN (2006). Generalized Additive Models: An Introduction with R.

[54] Hettmansperger TP, McKean JW (2010). Robust Nonparametric Statistical Methods.

[55] R Core Team (2022). R: A language and environment for statistical computing. 4.2.2 ed.

[56] Vancampfort D, Stubbs B, Mugisha J (2018). Physical activity and HIV in sub-Saharan Africa: a systematic review of correlates and levels. Afr Health Sci.

[57] Wright CH, Longenecker CT, Nazzindah R, Kityo C, Najjuuko T, Taylor K (2021). A Mixed Methods, Observational Investigation of Physical Activity, Exercise, and Diet Among Older Ugandans Living With and Without Chronic HIV Infection. J Assoc Nurses AIDS Care.

[58] Manne-Goehler J, Montana L, Gómez-Olivé FX, Rohr J, Harling G, Wagner RG (2017). The ART Advantage: Health Care Utilization for Diabetes and Hypertension in Rural South Africa. J Acquir Immune Defic Syndr.

[59] Yang L, Boily M-C, Rönn MM, Obiri-Yeboah D, Morhason-Bello I, Meda N (2023). Regional and country-level trends in cervical cancer screening coverage in sub-Saharan Africa: A systematic analysis of population-based surveys (2000–2020). PLoS Med.

[60] Yin RK (2009). Case Study Research: Design and Methods.

[61] Gomes-Neto M, Rodriguez I, Lédo AP, Vieira JPB, Brites C (2018). Muscle Strength and Aerobic Capacity in HIV-Infected Patients: A Systematic Review and Meta-Analysis. J Acquir Immune Defic Syndr.

[62] Hawkins KL, Brown TT, Margolick JB, Erlandson KM (2017). Geriatric syndromes: new frontiers in HIV and sarcopenia. Aids.

[63] Dalakas MC, Illa I, Pezeshkpour GH, Laukaitis JP, Cohen B, Griffin JL (1990). Mitochondrial myopathy caused by long-term zidovudine therapy. N Engl J Med.

[64] Johansson J, Strand BH, Morseth B, Hopstock LA, Grimsgaard S (2020). Differences in sarcopenia prevalence between upper-body and lower-body based EWGSOP2 muscle strength criteria: the Tromsø study 2015–2016. BMC Geriatr.

[65] Chetty L, Cobbing S, Chetty V (2021). Physical Activity and Exercise for Older People Living with HIV: A Scoping Review. HIV AIDS (Auckl).

[66] Siedner MJ (2019). Aging, Health, and Quality of Life for Older People Living With HIV in Sub-Saharan Africa: A Review and Proposed Conceptual Framework. J Aging Health.

[67] Schwartz CF, Sprangers MAG (2000). Adaptation to changing health: Response shift in quality of life research.

[68] Rueda S, Mitra S, Chen S, Gogolishvili D, Globerman J, Chambers L (2016). Examining the associations between HIV-related stigma and health outcomes in people living with HIV/AIDS: a series of meta-analyses. BMJ Open.

[69] Gupta A, Nadkarni G, Yang WT, Chandrasekhar A, Gupte N, Bisson GP (2011). Early mortality in adults initiating antiretroviral therapy (ART) in low- and middle-income countries (LMIC): a systematic review and meta-analysis. PLoS ONE.

