# Supplementary material for: The association between HIV infection, disability and lifestyle activity among middle-aged and older adults: an analytical cross-sectional study in Ivory Coast (the VIRAGE study)
Source: BMC Public Health. 2024 Jun 8;24:1549. doi: 10.1186/s12889-024-19020-9 (PMC11161960; doi:10.1186/s12889-024-19020-9)

**Supplementary Material**.

Functional tests

The short physical performance battery (SPPB) is a global functional test that combines the results of gait speed and chair stand and balance tests. For the SPPB balance subtest, participants are asked to stand holding their feet side by side for 10 seconds, then in semitandem and, eventually, in tandem position. In the chair stand subtest, the time to rise from a chair with arms across their chest and then sit for five repetitions is measured. The gait speed subtest is a 4-meter timed walk test at normal speed. Each component of the SPPB (gait, 5STS and balance subtest) is given a score between 0 and 4, and the sum of these scores results in the final SPPB score ranging from 0 to 12 (0 indicating the worst performance and 12 the highest degree of functioning).

The 6-minute walk test (6MWT) measures the distance covered in a period of 6 minutes of rapid walking on a flat and hard surface. It is a simple, global and practical test that assesses submaximal functional capacity. Participants choose their own intensity of exercise and are allowed to stop and rest. Therefore, the 6MWT provides information on the global and integrated responses of all systems involved during exercise (e.g., cardiovascular, pulmonary, neuromuscular) and reflects functional capacities for daily physical activities.

The five times sit-to-stand test (5STS) assesses lower limb power and speed. It measures the time required to complete 5 rapid sit-to-stand actions. Participants start sitting on a standard chair with knees close to 90° and feet comfortably placed. One or two practice sit-to-stand repetitions are allowed.

**Supplementary Figure 1.** International Classification of Functioning, Disability and Health (ICF) model with mapping to the information collected in the VIRAGE study.


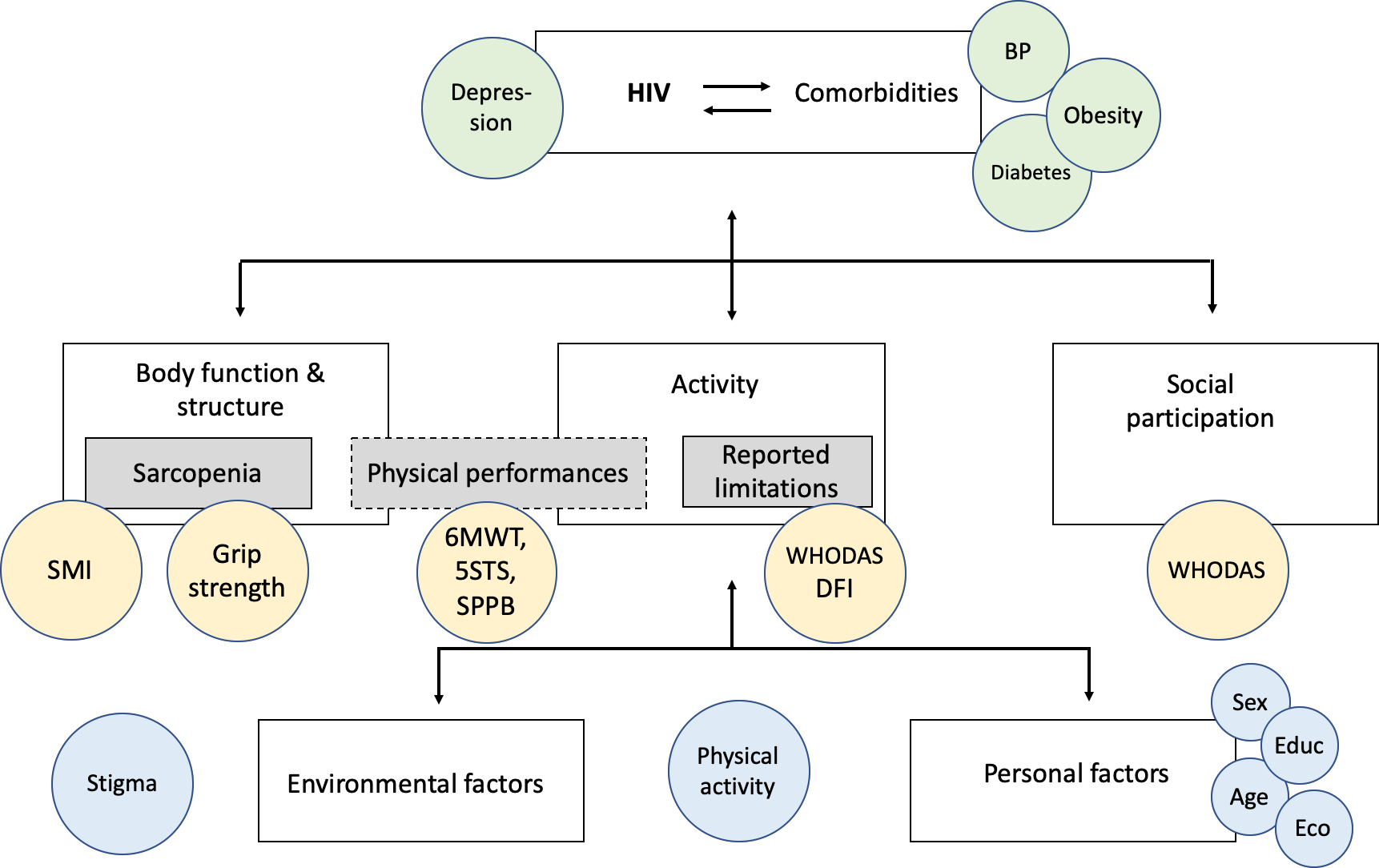

Supplement: Supplementary file 1 — Supplementary Material 1. [file 12889_2024_19020_MOESM1_ESM.docx]
